# In Silico and In Vitro Screening of Serine Racemase Agonist and In Vivo Efficacy on Alzheimer’s Disease *Drosophila melanogaster*

**DOI:** 10.3390/ph16020280

**Published:** 2023-02-13

**Authors:** Chih-Hao Lu, Hao-Teng Chang, Lee-Fen Hsu, Ming-Hsueh Lee, Jack Cheng, Dong Chuan Wu, Wei-Yong Lin

**Affiliations:** 1 Institute of Bioinformatics and Systems Biology, National Yang Ming Chiao Tung University, Hsinchu 300093, Taiwan; 2 Department of Biological Science and Technology, National Yang Ming Chiao Tung University, Hsinchu 300093, Taiwan; 3 Graduate Institute of Biomedical Sciences, China Medical University, Taichung 40402, Taiwan; 4 Department of Respiratory Care, Chang Gung University of Science and Technology, Puzi City 613, Chiayi County, Taiwan; 5 Chronic Disease and Health Promotion Research Center, Chang Gung University of Science and Technology, Puzi City 613, Chiayi County, Taiwan; 6 Division of Neurosurgery, Department of Surgery, Chang Gung Memorial Hospital, Puzi City 613, Chiayi County, Taiwan; 7 Graduate Institute of Integrated Medicine, China Medical University, Taichung 40402, Taiwan; 8 Department of Medical Research, China Medical University Hospital, Taichung 40402, Taiwan; 9 Translational Medicine Research Center, China Medical University Hospital, Taichung 40402, Taiwan; 10 Brain Diseases Research Center, China Medical University, Taichung 40402, Taiwan

**Keywords:** D-serine, serine racemase, NMDA receptor hypofunction, drug development

## Abstract

The NMDA receptor hypofunction has been implicated in schizophrenia, memory impairment, and Alzheimer’s disease. Modulating the abundance of D-serine, a co-agonist of the NMDA receptor, is a strategy to treat symptoms of the NMDA receptor hypofunction. In contrast to D-amino acid oxidase (DAAO) inhibitors, which aim at decreasing the loss of D-serine, this study tried to identify serine racemase (SRR) agonists, which boost the conversion of L-serine to D-serine. We used holo and apo structures of human SRR for the molecular docking against the National Cancer Institute (NCI) and ZINC compound databases and validated their efficacy by in vitro SRR activity assay. We identified NSC294149 (2-amino-3-(3-nitroimidazo[1,2-a]pyridin-2-yl)sulfanylpropanoic acid) as a potential SRR agonist and confirmed its amelioration of the hazard ratio of survival of the AD model of fruit fly (*Drosophila melanogaster*). These results suggest that the SRR agonist could be a drug design target against the NMDA receptor hypofunction symptoms.

## 1. Introduction

Serine racemase (SRR) is the major enzyme that converts L-serine to D-serine. The opening and closing of the ion channel of the NMDARs depend on both depolarization of the neuron cell, which repels the Mg^2+^ ions from the pore, and the ligand binding to binding sites at the extra-cellular domain of the NMDARs. In addition to glutamate, another glycine/D-serine has to bind to the NMDAR in conjunction to activate the receptor [1]. SRR knockout (SRR-/-) mice displayed an 85–90% reduction in D-serine level with alteration of NMDA neurotransmission, which decreased long-term potentiation [2]. In the SRR-mutant mouse, however, the decreased activation of the NMDA receptor and long-term potentiation can be recovered with the exogenous application of D-serine, suggesting the receptor supersensitivity [3]. In contrast, an abnormally high level of D-serine was demonstrated to be toxic to the central nervous system. The D-serine-induced NMDA excitotoxicity will cause postsynaptic neuron death in chronic neurodegenerative diseases and acute injuries such as ischemic stroke [3].

On the other hand, the NMDA receptor hypofunction (under excitation of the NMDA receptors) has been implicated in several diseases, including schizophrenia [4], psychosis [5], memory impairment [6], aging, and Alzheimer’s disease (AD) [7]. Specifically, NMDA receptor hypofunction is associated with the distribution of neurofibrillary tangles (NFTs) in AD [8]. Pharmachemical amelioration of the NMDA receptor hypofunction with second-generation antipsychotics (SGAs) could be used to treat schizophrenia. Although SGAs cause fewer extrapyramidal symptoms than first-generation antipsychotics, they target multiple neural receptors and cause metabolic side effects [9]. Therefore, an SRR agonist may boost the conversion of L-serine to D-serine and indirectly potentiate the NMDA receptor, and thus serve as a drug development strategy against the NMDA receptor hypofunction.

D-serine supplementation could prevent age-induced downregulation of NMDA receptor activity and rescue hippocampal synaptic plasticity [10,11,12,13]. The decline in D-serine in both aged rodent models [10,11,12] and human subjects [14] may explain the beneficial effects of D-serine supplementation. Interestingly, the decline in the D-serine could be attributed to a lower SRR expression both at transcriptional and translational levels, but not the enhancement of the D-serine catabolism by D-amino acid oxidase (DAAO) [10,11,12]. Although the Asc-1 transporter also determines the D-serine level around the synapse [15,16], the Asc-1 activity might be unchanged in the aged rodent hippocampus [17]. Thus, by excluding transportation and catabolism, insufficient D-serine synthesis by SRR could be the dominant cause of the decline in the D-serine in physiological aging.

Alzheimer’s disease is the most prevailing cause of abnormal cognitive deficit in the aged population [18]. Up to now, the pathological diagnosis of AD is the spread of neurofibrillary tangles (NFTs) of tau and the accumulation of amyloid beta (Aβ) [19]. In addition to memory deficit, motor function and psychiatric symptoms, including agitation, anxiety, and depression, are observed among AD patients [19]. Guided by the Aβ pathology hypothesis, efforts on therapeutic development have been concentrating on the inhibition of Aβ synthesis or the enhancement of Aβ clearance, such as the recent FDA-approved Aβ monoclonal antibody aducanumab [20], despite its controversial clinical outcome [21]. The difficulty of AD drug development drives the research community to tackle this problem from different aspects of the nature of AD, including the loss of neuroplasticity [22,23,24].

In the context of neuroplasticity, the NMDA receptor represents the dominant target for research [25,26], and the impairment of the NMDA receptor is hypothesized as the pivotal pathophysiological mechanism of AD [27,28,29]. For the two agonists of the NMDA receptor, the deficiency of the synaptic glutamate undisputedly advances AD pathology [30,31], while the change in the D-serine levels in AD remains controversial and might be model- and methodological-dependent. Biochemical investigations on the cerebrospinal fluid (CSF) or plasma changes in D-serine in AD appeared to be divergent in both magnitude and direction of change [32,33,34,35,36,37,38,39,40]. Similarly, AD mice models display divergent observations on the D-serine levels. On the one hand, D-serine levels increase in the hippocampal tissues of mice with intracerebroventricular injections of Aβ oligomers [38]. On the other hand, in 3xTg-AD mice, a decrease in L-serine synthesis, hippocampal neuroplasticity, and memory was observed, and these deficits could be rescued by long-term treatment of either L-serine or D-serine [41]. Le Douce and colleagues attributed the controversial D-serine levels in AD models to the fact that each AD model represents only partial aspects of and different stages of the pathological progression of AD [41]. One hypothesis is that D-serine levels in AD are in a “first down and then up” pattern in the progress of AD pathology [38], and the rise of the D-serine levels in the late stages might be due to the endogenous homeostasis to compensate for the hypofunction of the NMDA receptor.

Another issue is the benefit of SRR agonist treatment over direct D-serine administration. High doses of D-serine administration may cause proteinuria and asymptomatic transaminitis in human subjects [42], and long-term treatment of D-serine results in adverse side effects, including nephron- and hepato-toxicity [43,44]. On the contrary, the SRR agonist specifically affects tissues that express SRR, such as astrocytes in the CNS. Thus, fewer side effects are expected for SRR agonists than direct D-serine administration.

Therefore, this study aims to identify potential SRR agonists and prove the concept that SRR agonists could be therapeutics for diseases related to the hypofunction of the NMDA receptor due to the decrease in D-serine levels. 

This study used in silico docking and in vitro SRR activity assay to identify potential SRR agonists. Moreover, AD *Drosophila* was used as a model to test whether SRR agonist treatment from the early stage ameliorates AD symptoms. The AD model overexpresses human Aβ42 peptides in the nervous system and harbors degenerative neuronal phenotypes in a dose- and age-dependent manner [45], and the SRR agonist was treated as soon as the AD *Drosophila* emerged from the pupal case to simulate the early stage of AD, where the D-serine levels might be decreased.

## 2. Results

### 2.1. Site Moiety Map of Binding Pocket

**Figure 1:**
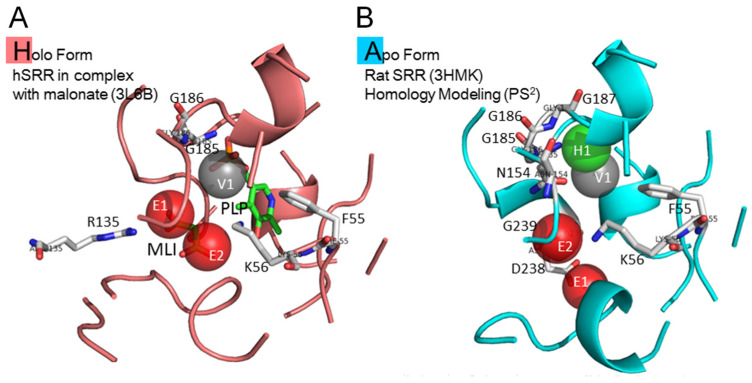
Three-dimensional schematic representations of SRR protein and the active binding sites. (**A**) The site moiety map of the holo form of human SRR and (**B**) of the apo form of rat SRR. MLI, malonate; PLP, vitamin B6 phosphate.

Both holo and apo structures of SRR were used as the target proteins for the molecular docking and post-screening analysis. Figure 1A,B show the interaction preference of holo and apo structures after being analyzed by SiMMap. Three anchors were found in the binding pocket of the holo structure, which were electronic moieties 1 (E1) and 2 (E2) and the van der Waals moiety (V1). The interaction residues in the binding pocket of the holo structure were arginine 135 for E1; lysine 56 for E2; and phenylalanine 55, glycine 185, and glycine 186 for V1. There were four anchors in the binding pocket of the apo structure, which were electronic moieties 1 (E1) and 2 (E2), the hydrogen bonding moiety (H1,), and the van der Waals moiety (V1). The interaction residues in the binding pocket were aspartic acid 238 for E1; lysine 56 for E2; asparagine 154, glycine 186, and glycine 187 for H1; and phenylalanine 55, glycine 185, and glycine 239 for V1. Compared with these two binding pockets of holo and apo structures, the interaction residues and binding moieties were arginine 135 for E1 in the holo form and asparagine 154, glycine 186, and glycine 187 for H1 in the apo form. It should be noted that the most important interaction residue and binding moiety was arginine 135 for E1 in the holo structure after binding with malonate.

### 2.2. Similar Compounds to Vitamin B6 Phosphate and Malonate

**Figure 2:**
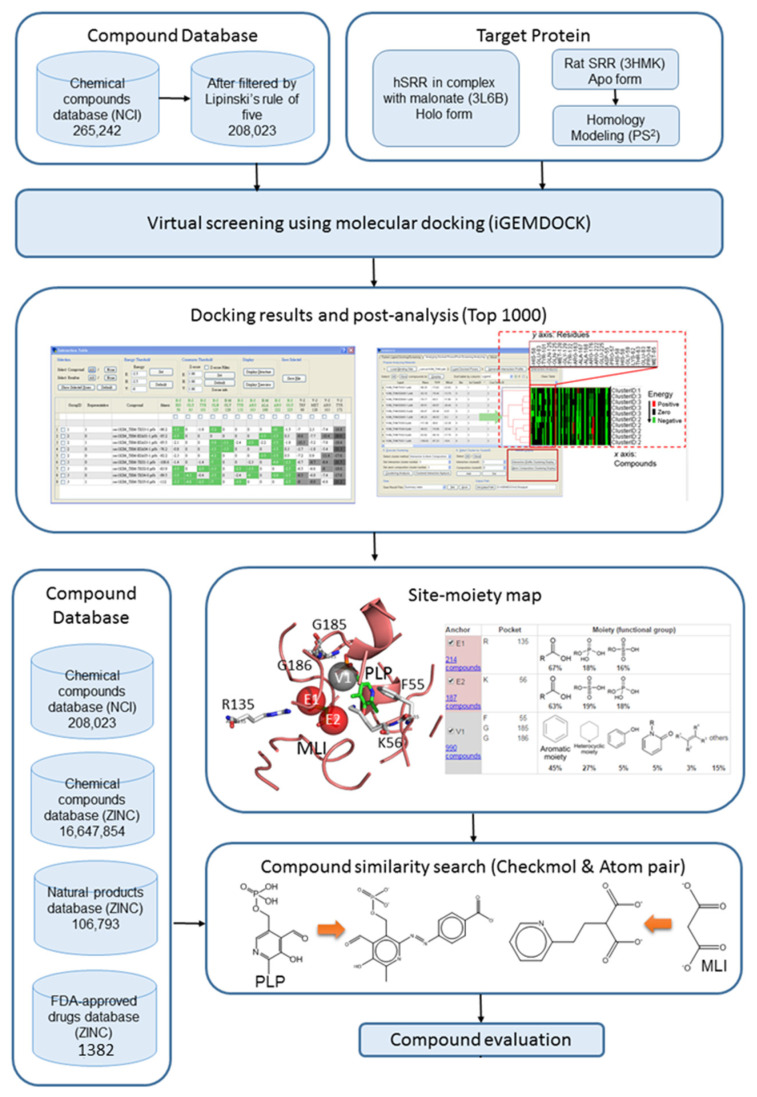
The work flow chart of this study.

After preparing the target protein structures, the compounds from the National Cancer Institute (NCI) and ZINC [46] databases were loaded to the molecular docking method, iGemdock [47], for virtual screening. In this step, 208,023 compounds from NCI and both holo and apo form structures of SRR were used. After screening, 1000 top-rank compounds and their docking poses were collected for post-analysis to determine the critical residues involved in the protein–ligand interaction in the binding pocket by SiMMap [48]. Three main interaction types were detected: van der Waals, electronic, and hydrogen bonding moieties for binding pockets. The flow chart is shown in Figure 2.

### 2.3. Similar Compounds to Vitamin B6 Phosphate and Malonate

**Figure 3:**
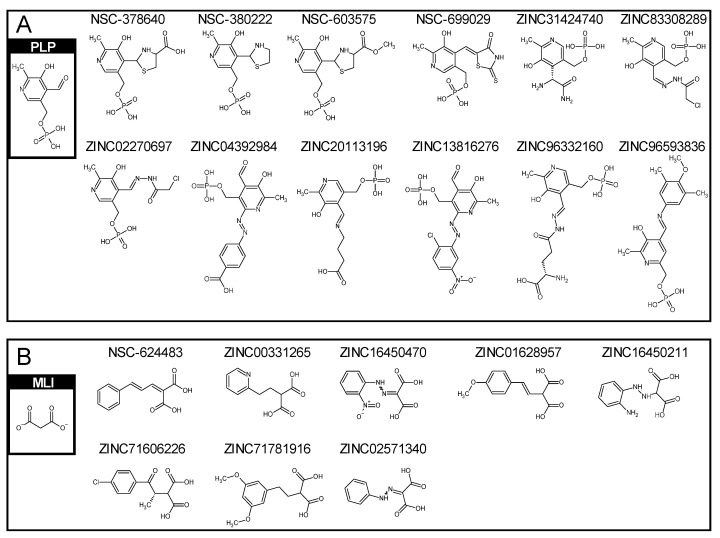
The compound IDs and their 2D structures of (**A**) PLP-like compounds and (**B**) MLI-like compounds.

Since the holo form SSR is in a complex with malonate (MLI) and vitamin B6 phosphate (PLP), in order to find the potential modulators which were similar to PLP and MLI, they were used individually as the core structures for the compound similarity search. After using Checkmol and Atom Pair approaches, there were twelve compounds similar to vitamin B6 phosphate, referred to as PLP-like compounds, which were four from the NCI database, seven from drug-like, one from natural products, and none from the FDA-approved drug database of ZINC. Correspondingly, there were eight MLI-like compounds, which were one from the NCI database, seven from drug-like, none from natural products, and none from the FDA-approved drug database of ZINC. The potential modulators are shown in Figure 3A,B after the compound similarity search using PLP and MLI as the core structures. The compound IDs and their 2D structures are also illustrated. Then, twelve PLP-like and eight MLI-like compounds were docked with the holo and apo structures of SRR by using iGemdock again. Moreover, in order to obtain more accurate docked results, the parameters we used in iGemdock were 800 for population, 80 for generation, and 10 for runs.

### 2.4. Top Candidates of Potential SRR Modulators

Twenty potential modulators were docked with the holo and apo structures of SRR by using iGemdock again, and their best-docked poses were examined carefully by combining the site moiety map from SiMMap. The compounds were ranked by using the SiMMap score, which combines the predicted binding energy from iGEMDOCK and the anchor scores between the map and the compound. Nine compounds were selected as the top-rank candidates of potential SRR modulators, four compounds for rank one, one for rank two, and four for rank three. One of these four compounds was collected from NCI, and three were from ZNIC, the compound IDs of which were NSC378640, ZINC20113196, ZINC96332160, and ZINC16450470, respectively. NSC378640, ZINC20113196, and ZINC96332160 were PLP-like compounds, and ZINC16450470 was an MLI-like compound. It should be noted that ZINC20113196 was also called GABA-pyridoxal phosphate, which was collected from the natural product database. Among these four compounds, the orientation was similar between the best-docked poses of holo and apo structures. These four compounds also had correct functional groups, which interacted with arginine 135 in the position of the E1 moiety of the holo form.

Another five compounds evaluated as ranks 2 and 3 were NSC699029, NSC624483, ZINC01628957, ZINC02571340, and ZINC71781916. NSC699029 was ranked two and was a PLP-like compound. NSC624483, ZINC01628957, ZINC02571340, and ZINC71781916 were ranked three and were MLI-like compounds. Among these five compounds, the orientation is not similar between the best-docked poses of holo and apo structures but had correct functional groups which interacted with arginine 135 in the position of the E1 moiety of the holo form.

### 2.5. Keeping PLP as a Cofactor Ligand

**Figure 4:**
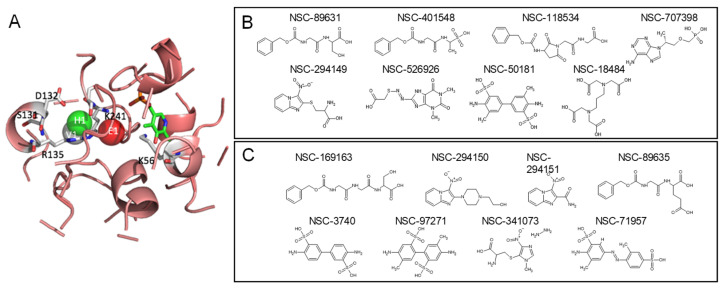
(**A**) The site moiety map of the holo form of SRR, which keeps the PLP as a cofactor ligand. (**B**) Potential modulators with PLP as a cofactor ligand. (**C**) Derivatives of potential modulators.

Another scheme was keeping the PLP as a cofactor ligand. In the results above, we focused on screening compounds that can replace MLI and PLP together. Here, the PLP was retained as a cofactor ligand in the holo structure of SRR, and 208,023 compounds from NCI were used for iGemdock. The parameters used in iGemdock were also 300 for population, 70 for generation, and 3 for runs. After screening, 1000 top-rank compounds and their docking poses were collected for post-analysis to determine the important residues involved in the protein–ligand interaction in the binding pocket by using SiMMap. Three anchors were found in the binding pocket of the holo structure, which were the electronic moiety (E1), hydrogen bond moiety (H1), and van der Waals moiety (V1) (Figure 4A). The interaction residues in the binding pocket of the holo structure were lysine 56 and arginine 135 for E1; glutamic acid 132, arginine 135, and serine for H1; and serine 131 and lysine 241 for V1. The most important interaction residue and binding moiety were lysine 56 and arginine 135 for E1.

In the top rank of 1000 compounds, 8 were involved in all three moieties’ interactions. The identifications of these eight potential compounds were NSC401548, NSC526926, NSC294149, NSC89631, NSC707398, NSC18484, NSC118534, and NSC50181 (Figure 4B). These eight compounds, as well as PLP-like or MLI-like compounds, were potential modulators of his. However, after asking for the price for these compounds from ZINC and NCI databases, there were only seven compounds that could be bought or requested, NSC18484, NSC50181, NSC89631, NSC118534, NSC294149, NSC378640, and NSC401548. Hence, we expanded the potential SRR modulators to include their derivatives, such as NSC3740, NSC97271, NSC71957, NSC169163, NSC89635, NSC341073, NSC294150, and NSC294151 (Figure 4C). These derivatives were requested and tested for in vitro SRR-modulating activity.

### 2.6. In Vitro Screening the SRR Modulators

**Figure 5:**
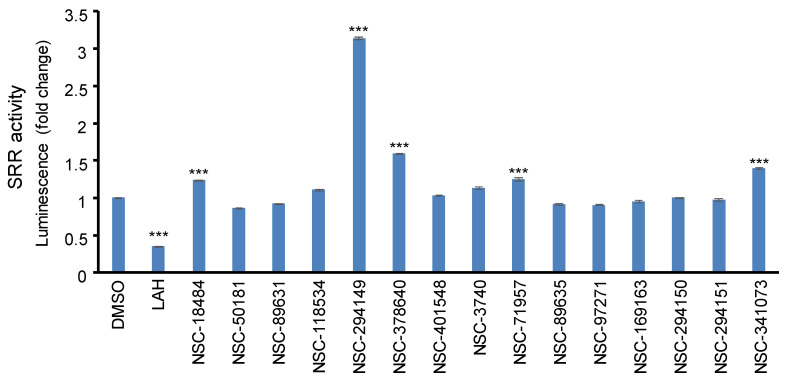
The effect of candidate compounds on SRR activity. DMSO serves as the blank control, while LAH is a positive control of the SRR inhibitor. The error bar is the standard error of the mean (SEM); *** denotes *p* < 0.0001 for Student’s *t*-test compared to DMSO control.

To evaluate the modulatory activity of the SRR modulator, 1.3 mM and 6.5 mM of LAH or sodium malonate was incubated with SRR enzymatic reaction. No matter 1.3 mM or 6.5 mM of LAH, the SRR activity was inhibited by more than 90%, but sodium malonate cannot inhibit SRR activity at the same concentrations. Moreover, LAH inhibited SRR activity in a dose-dependent manner, and the IC50 was roughly defined as 0.04 mM. The concentration was set as a standard concentration to compare with other novel compounds we identified. The candidate compounds were incubated with SRR enzymatic reactions, and the LAH at 0.04 mM was set as a positive inhibitory control. LAH inhibited around 40% of SRR activity, and the candidate compound NSC294149 significantly (*p* < 0.0001, Student’s *t*-test) increased by 200% of SRR activity (Figure 5).

### 2.7. The SRR Agonist NSC294149 Ameliorated Survival of AD Drosophila

**Figure 6:**
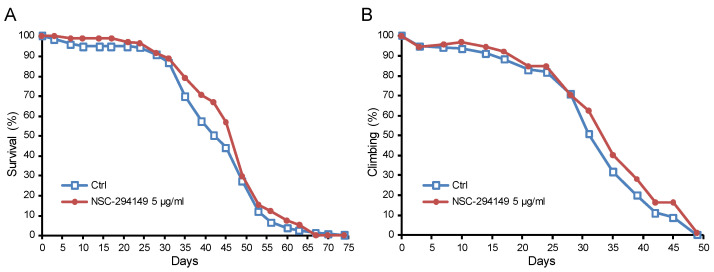
SRR agonist NSC294149 ameliorated (**A**) survival and (**B**) climbing ability of AD Drosophila.

As a proof of concept, the SRR agonist NSC294149 was applied to the Alzheimer’s disease (AD) fruit fly model (*Drosophila melanogaster*). In contrast to the untreated control group, NSC294149 treatment significantly lowered the hazard ratio of survival (0.7768, with a 95% CI of ratio 0.6021 to 1.002, Mantel–Haenszel method) (Figure 6A) and the climbing ability (0.8708, with 95% CI of ratio 0.7713 to 0.9832, Mantel–Haenszel method) (Figure 6B). 

## 3. Discussion

The NMDA receptor plays a critical role in CNS circuitry, and its hypofunction is a therapeutic target in schizophrenia, psychosis, and AD. However, since a potent agonist of the NMDA receptor may induce neurotoxicity due to calcium influx, alternative and milder NMDA receptor agonists might serve as a drug design strategy. Therefore, we focused on the NMDA receptor co-agonist D-serine and its upstream SRR, which converts L-serine to D-serine. By means of in silico docking and in vitro enzyme activity assay, we identified NSC294149 to be an SRR agonist. The following in vivo treatment showed that NSC294149 reduced the hazard ratio of survival of AD *Drosophila*.

As a co-agonist of the NMDA receptor, D-serine is indispensable for crucial physiological activities, including synaptic long-term potentiation (LTP) [1,49,50], spatial memory [51,52], prepulse inhibition of startle response [52], nociception [52], and even the relaxation of the corpus cavernosum [53]. The findings in this study could be applied to the abnormal physiological conditions due to D-serine deficiency in the described fields. Moreover, epidemiological evidence shows that serum levels of D-serine in AD patients were lower, and those of L-serine were higher than in normal controls [34]. The D-serine deficiency in AD may imply the hypofunction of the NMDA receptor and its dominating neuroplasticity and, thus, the downstream learning and memory. Notably, the lower D-serine to L-serine ratio in AD may imply the hypofunction of SRR and, thus, the opportunity for the SRR agonist as a therapeutic target.

Another field of application of the SRR agonist could be schizophrenia. The most determinate predictors of disability in schizophrenia are negative symptoms and cognitive impairments [54]. However, antipsychotic drugs targeting dopamine receptors have no effects on these predictors [54]. Epidemiological evidence shows that NMDA receptor hypofunction in the limbic brain region may explain behavioral abnormalities in schizophrenia [55,56,57]. With support from several lines of evidence from animal experiments [58,59], the NMDA receptor hypofunction hypothesis gains attention among the schizophrenia research community [60]. In the context of the NMDA receptor hypofunction hypothesis, the SRR agonist may serve as a therapeutic target for schizophrenia.

NSC294149, i.e., 2-amino-3-(3-nitroimidazo[1,2-a]pyridin-2-yl)sulfanylpropanoic acid, has a molecular weight of 282.28 and a topological polar surface area of 152 Å². According to the records of PubChem, NSC294149 has been tested for 70 BioAssays, including NCI human tumor cell line growth inhibition assay, NCI Yeast Anticancer Drug Screen, and eIF4E expression inhibitor, but none of them were active (https://pubchem.ncbi.nlm.nih.gov/compound/325414, accessed on 26 October 2018). 

There are some limitations on the interpretation of this study. First, we do not know whether NSC294149 acts on the SRR of *Drosophila*, and more broadly, we cannot assure whether NSC294149 acts on vertebrate SRR in vivo. Second, it is unclear whether NSC294149 penetrates the blood–brain barrier (BBB). Third, it is unclear whether NSC294149 specifically acts on SRR but not other neural receptors, resulting in the benefit of AD *Drosophila*. Fourth, we did not test whether NSC294149 bears side effects. Finally, we do not know whether NSC294149 benefits other NMDA receptor hypofunction symptoms, including schizophrenia and psychosis. However, these limitations do not falsify the conclusions and could be subjects of future research.

With the limitations, we are far from the translational application of NSC294149, even though this study at least showed the possibility that SRR agonists could serve as a strategy to develop the therapeutic target of the NMDA receptor hypofunction symptoms.

Other than the described limitations, the directions of future research on the SRR agonist include the following: 1. the comparison with D-serine for side effects, especially long-term or high-dosage treatment; 2. the effectiveness in other AD models induced by different genetic or chemical means; 3. the effectiveness in schizophrenia models to harbor evident hypofunction of the NMDA receptor; 4. the structural modification of the agonist to increase or even slightly decrease the SRR conversion activity as a target for drug development.

In conclusion, using in silico docking and in vitro screening, we identified NSC294149 (2-amino-3-(3-nitroimidazo[1,2-a]pyridin-2-yl)sulfanylpropanoic acid) as a potential SRR agonist, and its treatment on AD *Drosophila* improved the hazard ratio of survival and motor function. These results may imply that SRR agonists could be a drug design target against the NMDA receptor hypofunction symptoms.

## 4. Materials and Methods

### 4.1. Target Protein Structures

In this study, both holo and apo structures of serine racemase (SRR) were used as the target proteins for molecular docking. The holo form was the X-ray crystal structures of SSR in complex with malonate (MLI) and vitamin B6 phosphate (PLP) (PDB ID: 3L6B) [61], which was selected from the Protein Data Bank [62]. The vitamin B6 phosphate was nearby the malonate in the 3D coordinate space. Because there was no apo structure of human SSR in the Protein Data Bank, we used the homology modeling tool, PS^2^ [63], to generate it. PS^2^ is a protein structure prediction server using the homology modeling method. The apo form of rat SRR (PDB ID: 3HMK) [61] was used as the homology template. The sequence identity between human SRR and rat SRR was 91.85%, which was high enough to reflect the good quality of the predicted model for molecular docking. To understand the difference between the holo structure of SRR and predicted apo structure, these two structures were compared by CE [64], which is a structural alignment method.

### 4.2. Compound Databases

The databases used in this study were the compound databases of the National Cancer Institute (NCI) and ZINC [46]. ZINC is a free database of commercially available compounds for virtual screening provided by the Irwin and Shoichet Laboratories in the Department of Pharmaceutical Chemistry at the University of California, San Francisco (UCSF). The compound database of NCI contained 265,242 compounds and 208,023 after filtering by Lipinski’s rule of five [65]. In ZINC, the drug-like, natural products, and FDA-approved drug databases were used, which contained 16,647,854, 106,793, and 1382 compounds, respectively.

### 4.3. Molecular Docking and Post-Analysis

iGemdock [47] is a generic evolutionary method for molecular docking that computes a ligand conformation and orientation relative to the target protein’s active site. The parameters used in iGemdock were 300 for population, 70 for generation, and 3 for runs. SiMMap [48] was used for interaction preference recognition by analyzing the binding pocket with conserved interacting residues and specific physicochemical properties.

### 4.4. Compound Similarity Search

In this step, two approaches, Checkmol [48] and Atom Pair [66], were used for compound similarity search by recognizing the compound moieties and topological features. Checkmol is a program that can analyze the input molecule for the presence of various functional groups and structural elements. By the Atom Pair approach, compounds were clustered through hierarchical clustering based on their topological features. Then, four compound databases, the drug-like, natural products, and FDA-approved drugs in ZINC and NCI, were used for compound similarity search, which contained 16,647,854, 106,793, 1382 and 208,023 compounds, respectively.

### 4.5. The SRR Enzymatic Activity Assay

The commercial L-serine (Fluka, Morris Plains, NJ, USA) was treated with porcine D-amino acid oxidase (DAAO) (Sigma, St. Louis, MO, USA) to remove the D-serine contamination. Two hundred millimolar L-serine was mixed with 1.5 U/mL porcine DAAO, 3 U/mL catalase (Sigma, USA), and 84 μg/mL flavin adenine dinucleotide (FAD) (Sigma, USA) in 150 mM Tris buffer, pH 8.3. After a 16 h reaction, the DAAO was inactivated by heating to 95 °C for 10 min, and the debris was removed by centrifuge at 14,000× *g* for 10 min. The pretreated L-serine was stored at −20 °C before use. SRR activity was analyzed at 37 °C in a pH 8.0 reaction buffer containing 50 mM phosphate buffer, 15 μM pyridoxal-5′-phosphate, 1 mM MgCl_2_, 52 mM NaCl, 0.2 mM DTT, 1 mM ATP, and 10 mM pretreated L-serine. Then, 2.5 ug of SRR Enzyme Human Recombinant (ENZ-232, ProSpec, Rehovot, Israel) was added into the buffer and incubated at 37 °C for 10 min to analyze the racemization of L-serine to D-serine. The product D-serine was measured using the DAAO activity assay. Ten microliters of the mixture derived from the previous step was mixed with 100 mM Tris-HCl pH 8.8, 50 mM NaCl, horseradish peroxidase (HRP) (2 U/mL), 16 μM luminol, and 0.8 ug/mL FAD. After incubation at room temperature for 20 min, the luminescence was measured. The porcine DAAO (0.33 U/mL) was added to the reaction, and the luminescence was measured again immediately. The difference between the two luminescent values reflected the level of D-serine. The D-serine concentration can be fitted into the standard curve built with serial D-serine concentration. To measure the modulatory efficacy of candidate compounds, the compounds were mixed with SRR enzymatic reaction with the concentration indicated.

### 4.6. Fruit Fly Model of Alzheimer’s Disease

The AD fly model was produced by pan-neuronal expression of human amyloid-beta 42 protein as described previously [45]. Flies were maintained in a 12 h light–dark cycle at 25 °C. The culture medium formula was described previously [45]. The SRR modulating candidate was pre-diluted as 1 M DMSO solution and mixed into the culture medium before solidification to the final concentration as wished. The same amount of DMSO was added to the control medium. The treatment was given from the emergence of adult flies until their death. Only male flies were used, and more than 150 flies for each group were observed for their survival and climbing ability. The Gehan–Breslow–Wilcoxon test was used to estimate the significance of the difference between survival curves, and the Mantel–Haenszel method was used to estimate the hazard ratio.

## Data Availability

The data presented in this study are openly available in FigShare at https://doi.org/10.6084/m9.figshare.22032461, accessed on 2 February 2023.
